# Barriers to Incident Reporting by Physicians: A Survey of Surgical Residents and Attending Physicians

**DOI:** 10.7759/cureus.62850

**Published:** 2024-06-21

**Authors:** Madeline J Anderson, Wesley A Stephens, Brittany E Levy, Melissa R Newcomb, Andrew M Harris

**Affiliations:** 1 General Surgery, University of Kentucky, Lexington, USA; 2 Surgery, Lexington VA (Veterans Affairs) Medical Center, Lexington, USA

**Keywords:** zero harm, high reliability organization, near miss, adverse event, quality improvement, patient safety, incident reporting

## Abstract

Objectives

Incident reporting is vital to a culture of safety; however, physicians report at an alarmingly low rate. This study aimed to identify barriers to incident reporting among surgeons at a quaternary care center.

Methods

A survey was created utilizing components of the Agency for Healthcare Research and Quality (AHRQ) validated survey on patient safety culture. This tool was distributed to residents and attending physicians in general surgery and urology at a single academic medical center. Responses were de-identified and recorded for data analysis using REDCap (Research Electronic Data Capture) database tool (Vanderbilt University, Nashville, Tennessee, United States).

Results

We received 39 survey responses from 116 residents and attending physicians (34% response rate), including nine urologists and 30 general surgeons (24 attendings, 15 residents). Residents and attendings feel the person is being written up and not the issue (67%) and that there is a lack of feedback after changes are implemented (64%), though most believe adequate action is taken to address patient safety concerns (72%). Most do not report near-misses (64%), only significant adverse events (59%). Residents are likely to stay silent when patient safety events involve those in authority (60%). Faculty feel those in authority are open to patient safety concerns (67%), though residents feel neutral (47%) or disagree (33%).

Conclusion

Underreporting of incidents among physicians remains multifaceted and complex, from fear of retaliation to lack of feedback. Residents tend to feel less comfortable addressing authority figures when concerned about patient safety. While misunderstanding still exists about the applications and utility of incident reporting, a focus on quality over quantity could afford more meaningful progress toward high reliability in healthcare.

## Introduction

Prompted by the 1999 publication from the Institute of Medicine (IOM), To Err is Human, the use of incident reporting has been implemented across healthcare nationally to improve patient safety with the intent to reduce the number of adverse events and risk of preventable medical harm to patients [1]. The idea of utilizing incident reporting in healthcare for quality improvement was derived from its use in other high-risk industries such as aviation and nuclear power plants [1-4]. Deemed “high-reliability organizations” (HROs) for their potential for harm and low tolerance for error, these high-risk industries are invested in assuring safety and quality by following five important principles: preoccupation with failure, reluctance to simplify, sensitivity to operations, commitment to resilience, and deference to expertise [5-9].

However, despite implementing incident reporting, there is a significant lack of data to demonstrate or measure positive change within the field of quality improvement and patient safety in healthcare, and little has been done to advance the way incident reporting systems are structured and implemented [3,4,10]. A critical issue limiting potential in the quality of incident reports is the lack of physician engagement [4,11-14]. In a 2015 qualitative study by Mitchell et al., this was identified as one of five key issues limiting the success of the incident reporting system in healthcare, in addition to poor report processing, insufficient visible action, inadequate funding and institutional support, and inadequate use of evolving health information technology [4].

Our aim was to evaluate the utilization of and attitudes toward incident reporting among residents and attending physicians within the fields of general surgery and urology at a single academic institution and identify specific barriers to localized incident reporting. We hypothesized incident reporting rates among physicians at our institution would be poor and related to factors such as insufficient education regarding reportable events, fear of retaliation, and misunderstanding of the purpose of incident reporting.

## Materials and methods

This study was conducted at the University of Kentucky Healthcare, Lexington, Kentucky, United States, in September, 2022. The study was approved by the University of Kentucky Nonmedical Institutional Review Board (approval number: 63280).

Survey

A 20-question survey was created for completion utilizing components of the Agency for Healthcare Research and Quality (AHRQ)-validated survey on patient safety culture. The survey questions were designed to gain perspective on physician awareness and utilization of incident reporting and to better understand the current culture of incident reporting at our institution. An email invitation was sent to all general surgery and urology residency program trainees and attending physicians requesting voluntary participation in the study. The invitation detailed the scope and purpose of the study and outlined the importance of incident reporting for patient safety and quality improvement.

Survey Tool

The questionnaire had 20 questions in multiple-choice format. Eighteen questions used a 5-point Likert scale. Of these, options for responses to 16 of the questions included strongly agree, agree, neutral, disagree, and strongly disagree, and two questions were graded on a scale of never, rarely, sometimes, most of the time, and always. Of the remaining two questions, one asked the number of patient safety reports submitted within the past 12 months and the final question asked participants to select reasons for not submitting an incident report. The questions included in the survey are displayed in Table 1.

**Table 1:** The 20 questions included in the survey sent out to residents and attending physicians within the general surgery and urology departments at our institution.

	Question	Response
1	When an event is reported in the department, it feels like the person is being written up, not the problem.	5-point Likert
2	In the department, there is a lack of support for staff involved in patient safety errors.	5-point Likert
3	When staff make errors, the department focuses on learning rather than blaming individuals.	5-point Likert
4	In the department, staff speak up if they see something that may negatively affect patient care.	5-point Likert
5	When staff in this department see someone with more authority doing something unsafe for patients, they feel comfortable speaking up.	5-point Likert
6	When the staff in this department speak up, those with more authority are open to their patient safety concerns.	5-point Likert
7	My supervisor takes action to address patient safety concerns that are brought to their attention.	5-point Likert
8	In the department, changes to improve patient safety are evaluated to see how well they worked.	5-point Likert
9	In this department, we are informed about changes that are made based on event reports.	5-point Likert
10	The department lets the same patient safety problems keep happening.	5-point Likert
11	When errors happen in the department, we discuss ways to prevent them from happening again.	5-point Likert
12	Leaders within the department examine near-miss events that could have harmed patients but did not.	5-point Likert
13	Management seems interested in patient safety only after an adverse event happens.	5-point Likert
14	Just before the start of procedures, all team members stopped to discuss the overall plan of what was to be done.	5-point Likert
15	Just before the start of procedures, the team was encouraged to speak up at any time if they had any concerns.	5-point Likert
16	Immediately after procedures, team members discussed any concerns for patient recovery.	5-point Likert
17	When a mistake is caught and corrected before reaching the patient, how often is this reported?	5-point Likert
18	When a mistake reaches the patient and could have harmed the patient, but did not how often is that reported?	5-point Likert
19	In the past 12 months, how many patient safety reports have you reported?	A) 1-2 B) 3-5 C) 6-10 D) 11+
20	I don’t report incidents because (select all that apply):	I do not know which incidents should be reported. I typically report only significant adverse events. I become too busy and forget to make a report. Adverse incident reporting has little impact on the quality of care. I have not received proper feedback or noticed improvement from previous reports. I am worried about disciplinary action within my department. I am worried about litigation. I don’t know whose responsibility it is to make the report. My colleagues may be unsupportive as the case is dealt with. I do not want the case discussed in formal settings. The circumstances or outcomes of the case make reporting unnecessary. As long as the staff involved learn from the incidents, it is unnecessary to discuss them further. Junior staff are often unfairly blamed for adverse incidents. It takes too long or is too cumbersome to fill out the report.

Participants

Inclusion criteria for this study comprised all resident physicians and attending physicians within the departments of general surgery and urology at our institution who chose to respond to the survey and answered most survey questions. Of the 39 survey responses included in this study, there were eight incomplete survey responses with between one and six unanswered questions, including three resident and five attending physician responses. The incomplete survey responses were reported and factored into percentage calculations.

Residents or attending physicians outside the departments of general surgery and urology at our institution or physicians within these departments who chose not to respond to the survey were excluded. Additionally, of the survey responses received, three respondents were excluded from the study due to answering only two or fewer questions, including two residents and one attending physician.

Data collection and storage

Survey responses were collected, de-identified, and stored securely in a REDCap (Research Electronic Data Capture) database (Vanderbilt University, Nashville, Tennessee, United States) for analysis.

## Results

Survey responses

**Figure 1:**
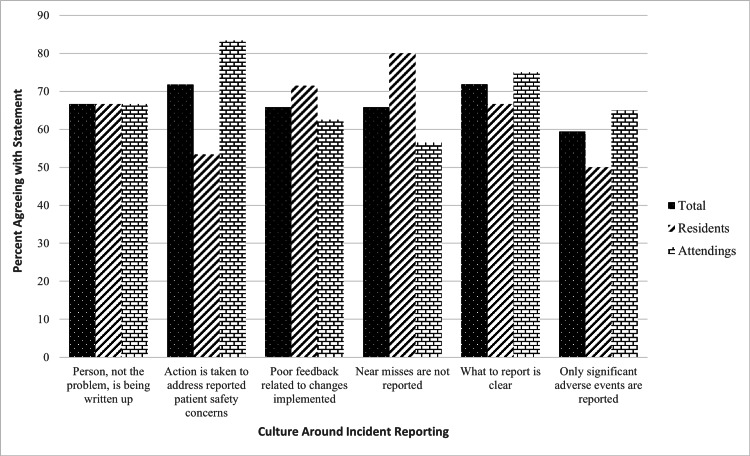
Six common perceptions of incident reporting among residents and attending physicians, displaying overall percentage of physicians agreeing and the breakdown between residents and attendings.

A total of 116 trainees were invited to participate in the study; of these, 39 participants (34% response rate) submitted complete survey responses. Responses were received from 24 attending physicians, including 18 general surgeons and six urologists, as well as 15 resident physicians, including 12 general surgery residents and three urology residents. This resulted in 20 responses from the general surgery department and nine from the urology department (Figure 1).

Person or Problem

Overall, it was found that 10 of 15 (67%) residents and 16 of 24 (67%) attendings agreed that when an incident is reported, it feels as if the person, rather than the problem, is being written up.

Lack of Feedback

When an incident report is submitted, 10 of 15 (67%) residents and 15 of 23 (65%) attendings agreed that there is poor feedback related to the changes implemented in response to incident reporting. However, most resident physicians (eight of 15, 53%) and attendings (20 of 24, 83%) agreed that they believed that action is taken by supervisors to address reported patient safety concerns (72% of participants overall), though there is higher overall confidence from attendings than residents.

Near Misses vs Adverse Events

When comparing patient safety incidents resulting in adverse events vs near-misses, both residents (12 of 15, 80%) and attendings (13 of 23, 57%) agreed near-miss events were never, rarely, or only sometimes reported; Additionally, more than half of residents (six of 12, 50%) and attendings (13 of 20, 65%) agreed they typically only report patient safety incidents resulting in significant adverse events (64% and 59%, respectively of participants overall), though seven of 39 participants did not complete this question of the survey.

Number of Reports Submitted

Over the past 12 months, six of 13 (46%) residents and seven of 24 (29%) attendings had submitted at least one incident report, with an average number of 1.3 incident reports filed per resident overall and 1.2 incidents per attending overall; more residents filed at least one incident report within a year, and though fewer overall attendings filed any incident reports, the attendings who did file incident reports filed a higher number of reports overall within the past year. 

Perspective on Authority

Nine of 15 (60%) residents disagreed that staff felt comfortable speaking up when seeing someone in authority do something unsafe for patients; however, attendings felt more neutral about this, with only 10 of 14 (42%) attendings disagreeing. Furthermore, 16 of 24 (67%) attendings felt that those in authority were open to patient safety concerns from staff (67%) while only three out of 15 (20%) residents agreed.

## Discussion

Physician engagement in reporting patient safety events remains poor, despite over 20 years of implementing incident reporting systems in healthcare. Survey responses to a 20-question survey from 24 attending surgeons and 15 resident surgeons within the departments of general surgery and urology at our institution were evaluated to better understand physician engagement, barriers to physician reporting, and the current culture surrounding incident reporting at our institution. With more insight at the institutional level, there are opportunities for re-education, to redirect the focus to quality over quantity, with the intent of evaluating true patient safety events to reduce harm rather than a focus on interpersonal issues and punitive action.

Incident reporting in healthcare was implemented to improve patient safety by identifying specific patient safety events to investigate, and then reflecting on potential system changes to be made to reduce the risk of similar adverse events occurring in the future [1,4-9,12,15]. HROs such as aviation, from where our system was adopted, rely heavily on their existing culture of inquiry, investigation, and improvement to successfully achieve meaningful reduction in adverse events [1,3,4,10].

Mistranslation of incident reporting from other HROs to healthcare is evident in the inadequate triaging of reports and lack of subsequent investigation into change-evoking events [4,16]. While continued reporting of all patient safety events, including near misses, is encouraged to maximize opportunities to learn from these events to prevent patient harm, misunderstanding of what constitutes a patient safety event can lead to over-reporting of work conflicts that have little to do with patient safety. The focus on quantity over quality of reports submitted and inadequate infrastructure to process a high volume of reports delays appropriate, timely responses to true patient safety events [3,4,15]. Additionally, the lack of closed-loop communication and visible action continue to limit motivation to report, especially among physicians. These themes of closed-loop communication were echoed in this study’s results in which 67% of residents and 65% of attendings felt they did not receive feedback regarding the reported event and therefore may be less likely to report in the future [3,4,14]. Furthermore, healthcare is unique from other HROs in its high variability related to staffing as well as individual patients and clinical scenarios [3]. This makes the universal application of solutions to patient safety events and standardization of protocols challenging.

In a qualitative study by Mitchell et al., 11 international patient safety experts were interviewed to identify five key issues limiting the utility of incident reporting systems over the prior 15 years [4]. These challenges were defined as poor report processing (due to the high volume of reports with inadequate triaging of high-yield patient safety incidents for system learning), lack of physician engagement, insufficient visible action (including lack of feedback), inadequate funding and institutional support, and inadequate use of evolving health information technology [4].

Lack of physician engagement limits the future growth and success of this endeavor [3,4]. There continues to be an exceptionally low percentage of reports submitted by physicians [4,13,14,17], as was reproduced in our study in which only 46% of residents and 29% of attendings reported having submitted an incident report in the past 12 months. Most incident reports are submitted by nursing staff, rather than physicians. This is likely multifactorial, related to issues including physician workload, lack of instruction on what types of events should be reported, lack of feedback on reports submitted, medicolegal fear, and lack of meaningful evidence demonstrating the efficacy of incident reporting in patient safety improvement [2-4]. In our survey, leading reasons included lack of feedback, misunderstanding of what should be reported, and fear of reporting those in authority. 

Incident reports submitted by physicians offer valuable perspectives on medical errors and a broad view of patient safety events, given the critical role that physicians play in patient care. For this reason, several studies have attempted to increase the proportion of incident reports submitted by physicians using various tactics including financial incentives, routine patient safety rounds, small group education, and feedback from physician leadership [13,14,17,18]. We found physicians at our institution underreported for reasons including feeling as if people rather than events were being written up, hesitation when reporting events involving those in authority, and misunderstanding of what types of patient safety events should be reported. Respondents also reported a lack of feedback on previously submitted reports; however, most trusted that action had been taken despite this.

Personal attack or a systems issue?

One of the drawbacks of incident reporting in the current healthcare culture is the tendency to focus on assigning fault to the staff member involved in the event rather than investigating the event itself or analyzing the root cause [3-5,10,19]. Our survey of residents and attending physicians in general surgery and urology reflected this trend, with both groups agreeing (67%) that incident reports feel like the person is being written up rather than the problem.

This suggests a lack of awareness of the purpose of incident reporting. It also conveys the physicians involved in this study expect a reactionary or disciplinary response to the incident reports submitted, rather than a systems improvement resulting from comprehensive inquiry and investigation.

To achieve meaningful change and improve patient safety, analysis of incident reports should prompt a thorough investigation of the organizational factors at play and the opportunities for system improvement rather than focusing on the individual and assigning blame or punishment [6,12,15,16,19]. More effort must be made to change the culture toward collective interest in quality improvement and patient safety, rather than focusing on disciplinary action, remediation, and retaliation [3,4,6,12,16].

Impact of hierarchy: fear of reporting errors from those in authority, lack of trust in authority

The perception of hierarchy in medicine significantly impacts fear of retaliation and is another factor limiting the incident reporting system in healthcare. Out of all respondents to our survey, 60% of residents did not think staff felt comfortable speaking up when seeing someone in authority do something unsafe for patients, though attending respondents took a more neutral stance. Additionally, only 20% of resident respondents felt those in authority were open to patient safety concerns from staff.

It is important all members of the healthcare team feel comfortable submitting incident reports so we can continue to investigate patient safety events. Unfortunately, when investigating a reported event, it can become difficult to maintain the anonymity of the reporting or involved staff. This Catch-22 hinders staff from reporting incidents involving those in authority. It is also critical to maintain the confidentiality of the patient; incident reporting has not yet been approved for use within patients' electronic medical records (EMRs), though utilizing EMRs for data collection could potentially afford opportunities to analyze a larger repository of data.

Inadequate feedback: belief in action despite lack of feedback

Another challenge identified in the current incident reporting system in healthcare is the lack of visible action and quality feedback, which may further deter physicians from submitting incident reports [10,11,14,20]. Despite 67% of residents and 65% of attending physicians surveyed in this study reporting lack of feedback, about 72% of participants agree they believe appropriate action is taken by supervisors to address the incident reports submitted. However, this also means over one-third of physicians surveyed do not think appropriate action is taken.

A study published by Ngo et al. in 2022 used a controlled before-and-after design to investigate why physicians report patient safety events so infrequently [14]. Based on a focus group discussion, they implemented a feedback system for physician-submitted reports in which a physician responder would provide feedback within one week of the submission that addressed the incident and the resultant response. By doing so, they were able to double the percentage of incident reports submitted by physicians [14]. This suggests by providing consistent feedback and timely updates on the actions taken in response to the incident, physicians may be more likely to report patient safety events.

Reporting adverse events but not near misses

Patient safety events include both near misses and adverse events. Whether or not a patient is harmed by an event, there is an opportunity to learn from the mistakes of others through a more thorough investigation of the event [1,18,19]. This ties into one of the five principles of an HRO: preoccupation with failure [5-9]. It is important to examine both near failure and failure to better understand the problems that exist within the system to avoid failure the next time a similar event occurs. However, misconceptions about what should and should not be reported persist. Both 80% of residents and 57% of attending physician respondents to our survey reported low likelihood of submitting near-miss events, and over half of respondents said that they typically only reported incidents that result in adverse events.

Underreporting remains a significant issue in the United States, with approximately 50-96% of patient safety events estimated to not be reported [2,21,22]. One potential reason for underreporting is a lack of education on what type of events should be reported. In a study by Krouss et al. involving 73 residents, reporting increased from one to 10 reports submitted monthly over the course of six months by implementing patient safety rounds, which included educating participants on how and what to submit as well as providing a reference card to re-enforce this education [13]. Results of this survey suggest opportunities for re-education, with the intent of improving the quality of reports submitted and a renewed focus on patient safety rather than interpersonal conflict.

Next steps

The results of our survey echo much of the literature regarding poor physician engagement and the overall challenges faced with incident reporting systems in healthcare as they exist today. While misunderstanding still exists about the applications and utility of incident reporting, a focus on quality over quantity could afford more meaningful progress toward high reliability in healthcare. To maintain physician engagement, it is important that consistent, timely, and quality feedback is provided in response to submitted incident reports. Additionally, redirection of focus on critical patient safety events rather than interpersonal conflict and blame-casting is crucial to the success of this process.

More research is needed to quantify the risk reduction for adverse events to provide evidence of the value of incident reporting in healthcare. Many studies have been published to examine the current culture and usage of incident reporting; however little research has been done to quantify the impact of incident reporting on reducing adverse events. With a renewed focus on quality and intent at the institutional level, incident reporting systems could become successful in healthcare as they have in other high-reliability industries.

Limitations

This study was limited by a small sample size, with 39 participants, exclusively within general surgery and urology departments, with 15 resident participants. The 34% response rate to the survey limits the results and may not accurately represent the views of the whole targeted population. Additionally, the survey was not fully completed by eight respondents, limiting data to the last one to six questions. The survey was designed in question-and-answer format, though more nuanced responses may have been elicited in a more open-ended setting such as a focus group.

## Conclusions

Poor physician engagement in incident reporting has been identified as one of the key factors limiting the success of incident reporting systems in healthcare. Underreporting among both residents and attending physicians is multifactorial, with key issues continuing to be a lack of timely feedback and visible action, a lack of understanding of what incidents should be reported, fear of reporting those in authority, and fear of retaliation. While misunderstanding regarding the applications and utility of incident reporting persists, there is an opportunity for a renewed focus on true patient safety events as well as quality over quantity at the institutional level to make meaningful progress toward high reliability in healthcare.
